# First report of Seville root-knot nematode, *Meloidogyne hispanica* (Nematoda: Meloidogynidae) in the USA and North America

**DOI:** 10.21307/jofnem-2021-098

**Published:** 2021-12-01

**Authors:** Andrea M. Skantar, Zafar A. Handoo, Sergei A. Subbotin, Mihail R. Kantor, Paulo Vieira, Paula Agudelo, Maria N. Hult, Stephen Rogers

**Affiliations:** 1 Mycology and Nematology Genetic Diversity and Biology Laboratory, USDA, ARS, Northeast Area, Beltsville, MD, 20705; 2 Plant Pest Diagnostic Center, California Department of Food and Agriculture, 3294 Meadowview Road, Sacramento, CA, 95832; 3 Center of Parasitology of A.N. Severtsov Institute of Ecology and Evolution of the Russian Academy of Sciences, Leninskii Prospect 33, Moscow, 117071, Russia; 4 USDA-ARS, Molecular Plant Pathology Laboratory, Beltsville, MD, 20705-2350; 5 School of Plant and Environmental Science, Virginia Tech, Blacksburg, VA, 24061; 6 School of Agricultural, Forest, and Environmental Sciences, Clemson University, Clemson, SC, 29634

**Keywords:** Corn, Meloidogyne hispanica, North America

## Abstract

A high number of second stage juveniles of the root-knot nematode were recovered from soil samples collected from a corn field, located in Pickens County, South Carolina, USA in 2019. Extracted nematodes were examined morphologically and molecularly for species identification which indicated that the specimens of root knot juveniles were *Meloidogyne hispanica.* The morphological examination and morphometric details from second-stage juveniles were consistent with the original description and redescriptions of this species. The ITS rRNA, D2-D3 expansion segments of 28S rRNA, intergenic *COII*-16S region, *nad5* and *COI* gene sequences were obtained from the South Carolina population of *M. hispanica*. Phylogenetic analysis of the intergenic *COII*-16S region of mtDNA gene sequence alignment using statistical parsimony showed that the South Carolina population clustered with *Meloidogyne hispanica* from Portugal and Australia. To our best knowledge, this finding represents the first report of *Meloidogyne hispanica* in the USA and North America.

According to the USDA’s National Agricultural Statistics Service (NASS), corn was the largest crop in America in 2019 with 91.7 million acres planted (USDA, National Agricultural Statistics Service, 2019a). Corn was also the most planted crop in South Carolina, with 380,000 acres planted in 2019 (USDA, National Agricultural Statistics Service, 2019b). Corn has many known pests and the most common nematodes affecting this valuable crop are spiral and root-lesion nematodes, followed by dagger, needle, ring, stunt, pin, lance, and stubby root nematodes (Tylka et al., 2011; Yan et al., 2016).

The Seville root-knot nematode, *Meloidogyne hispanica* (Hirschmann, 1986) was studied for the first time by Dalmasso and Bergé (1978) from Seville, Spain from peach rootstock (*Prunus persica silvestris* Batsch) and later described as *M. hispanica* by Hirschmann (1986). The species has been reported infecting many economically important crops such as tomato, beet, corn, pepper, cucumber, eggplant, potato, bean, and others in several countries of Europe, Asia, Africa, Central and South America (Maleita et al., 2012a, 2012b; Subbotin et al., 2021). Until now, there have been no verified reports of this nematode in North America. *Meloidogyne hispanica* belongs to the *Ethiopica* group of root-knot nematodes, which also contains the species *M. ethiopica, M. luci* and *M. inornata* (Alvarez-Ortega et al., 2019).

The objective of this work was to provide morphological and molecular characterization of this root-knot nematode isolated from corn in South Carolina, identified herein as *Meloidogyne hispanica.* This report represents the first record of this species in the USA and North America.

## Materials and methods

Two soil samples collected from a corn field from Pickens County, South Carolina were sent by Diana Low (Clemson University) to the Mycology and Nematology Genetic Diversity and Biology Laboratory (MNGDBL), Beltsville, MD in fall of 2019 and early 2020. Nematodes were extracted from soil using sugar centrifugal flotation method (Jenkins, 1968). For morphological study nematodes were fixed in 3% formaldehyde and processed to glycerin by the formalin glycerin method (Hooper, 1970; Golden, 1990). Photomicrographs of the specimens were made with a Nikon Eclipse Ni compound microscope using a Nikon DS-Ri2 camera. Measurements were made with an ocular micrometer on a Leica WILD MPS48, Leitz DMRB compound microscope.

For molecular identifications, single nematodes were mechanically disrupted with a micro knife in 20  µl nematode extraction buffer (500  mM KCl, 100  mM Tris-Cl (pH8.3), 15  mM MgCl_2_, 10  mM dithiothreitol (DTT), 4.5% Tween 20 and 0.1% gelatin) (Thomas et al., 1997) and stored at −80°C until needed. To prepare DNA extract, frozen nematodes were thawed, 1 µl proteinase K (from 2 mg/ml stock solution) was added, and the tubes were incubated at 60°C for 60 min, followed by 95°C for 15 min to deactivate the proteinase K. Two or three microliters of extract were used for each PCR reaction. Six J2 were examined for each marker.

DNA markers were amplified using the following primers: the internal transcribed spacer region (ITS1-5.8S-ITS2) of rRNA gene was amplified with primers TW81 [5′-GTTTCCGTAGGTGAACCTGC-3′] and AB28 [5′-ATATGCTTAAGTTCAGCGGGT-3′] as described by Skantar et al. (2012); the D2–D3 expansion segments of the large subunit (LSU) 28S rRNA gene were amplified with primers D2A [5′- ACAAGTACCGTGAGGGAAAGTT-3′] and D3B [5′-TCGGAAGGAACCAGCTACTA-3′] according to De Ley et al. (2005). The mitochondrial *nad5* region was amplified with primers NAD5F2 [5′-TATTTTTTGTTTGAGATATATTAG-3′] and NAD5R1 [5′-TATTTTTTGTTTGAGATATATTAG-3′] as described (Janssen et al., 2016); the mtDNA intergenic *COII*-16S region was amplified with primers C2F3 [5′-GGTCAATGTTCAGAAATTTGTGG-3′] and 1108 [5′-TACCTTTGACCAATCACGCT-3′] as described by Powers and Harris (1993); and mitochondrial *COI* was amplified with primers JB3 [5′-TTTTTTGGGCATCCTGAGGTTTAT-3′] and JB5 [5′-AGCACCTAAACTTAAAACATAATGAAAATG-3′] according to Derycke et al. (2010).

All PCR products were cleaned with the Monarch DNA Gel Extraction Kit (NEB, Ipswitch, MA). The ITS rRNA gene fragments were cloned using the Strataclone PCR Cloning Kit (Agilent, Santa Clara, CA); cleaned amplicons were sequenced directly by Genewiz, Inc. Cloned plasmid DNA was prepared with the Monarch Plasmid Miniprep Kit (NEB) and sequenced. Distinct sequences obtained from as many as six J2 were submitted to GenBank as follows: *M. hispanica:* 28S rRNA gene, MZ328884, MZ328885; *COI* gene, MZ332972-MZ332973; *nad5* gene, MZ332520-MZ332521; *COII-16S*, region MZ332519; ITS clone sequences came from specimens 112C5 (MZ328463, MZ328455), 112C6 (MZ328459, MZ328461), 112C7 (MZ328456, MZ328457), 112E1 (MZ328452, MZ328460), 112E2 (MZ328458, MZ328454), 112E3 (MZ328453, MZ328462).

Alignments with the ITS rRNA, D2-D3 of 28S rRNA, *COI*, nad5 gene sequences and intergenic *COII*-16S gene region of mtDNA of new sequences *M hispanica* with other sequences of this species and other root-knot nematodes were created using ClustalX 1.83 (Chenna et al., 2003) with default parameters. The alignments for ITS rRNA, D2-D3 of 28S rRNAand *COII-*16S gene sequences were used to construct phylogenetic networks using statistical parsimony (SP) as implemented in POPART software (http://popart.otago.ac.nz) (Bandelt et al., 1999).

## Results and discussion

### Meloidogyne hispanica measurements

*Second stage juveniles* (*n*  =  10) L = 342.0 ± 14.6 (325.0–352.0)  μm, stylet = 10.5 ± 0.6 (10.0–11.5)  μm, tail length = 41.7 ± 1.2 (40.0–43.0)  μm; hyaline region = 12.8 ± 1.5 (10.0 –15.0)  μm; *a*  =  26.8 ± 1.4 (25.0–29.0); *b*  =  2.9 ± 0.1 (2.7–3.2); *c* = 8.3 ± 0.5 (7.8–9.4).

### Meloidogyne hispanica descriptions

Second-stage juveniles (Fig, 1). Body annules distinct, becoming irregular in posterior tail region. Four incisures in lateral field, outer lines crenate. Labial region truncate, offset from body. Stylet delicate, with cone sharply pointed, increasing in width posteriorly, shaft cylindrical, widens slightly posteriorly, knobs robust, distinctly separated, rounded, and sloping posteriorly. Pharyngo-intestinal junction indistinct, near nerve ring. Pharyngeal gland lobe variable in length. Hemizonid 2 annuli anterior to excretory pore. Tail slender, with a bluntly rounded terminus. Posterior tail region, with large annules of variable size. Hyaline region indistinct. Rectal dilation large. Phasmids obscure but located a short distance posterior to anal opening. The body mean length measurements for this isolate are shorter from the original description 392.0 (356.4–441.4) μm. The mean values of the stylet length and tail length are also shorter from the population described by Hirschmann (1986) but still within the range. All other morphology and morphometrics of the South Carolina population fit very well with the type specimens from the original description.

**Figure 1:**
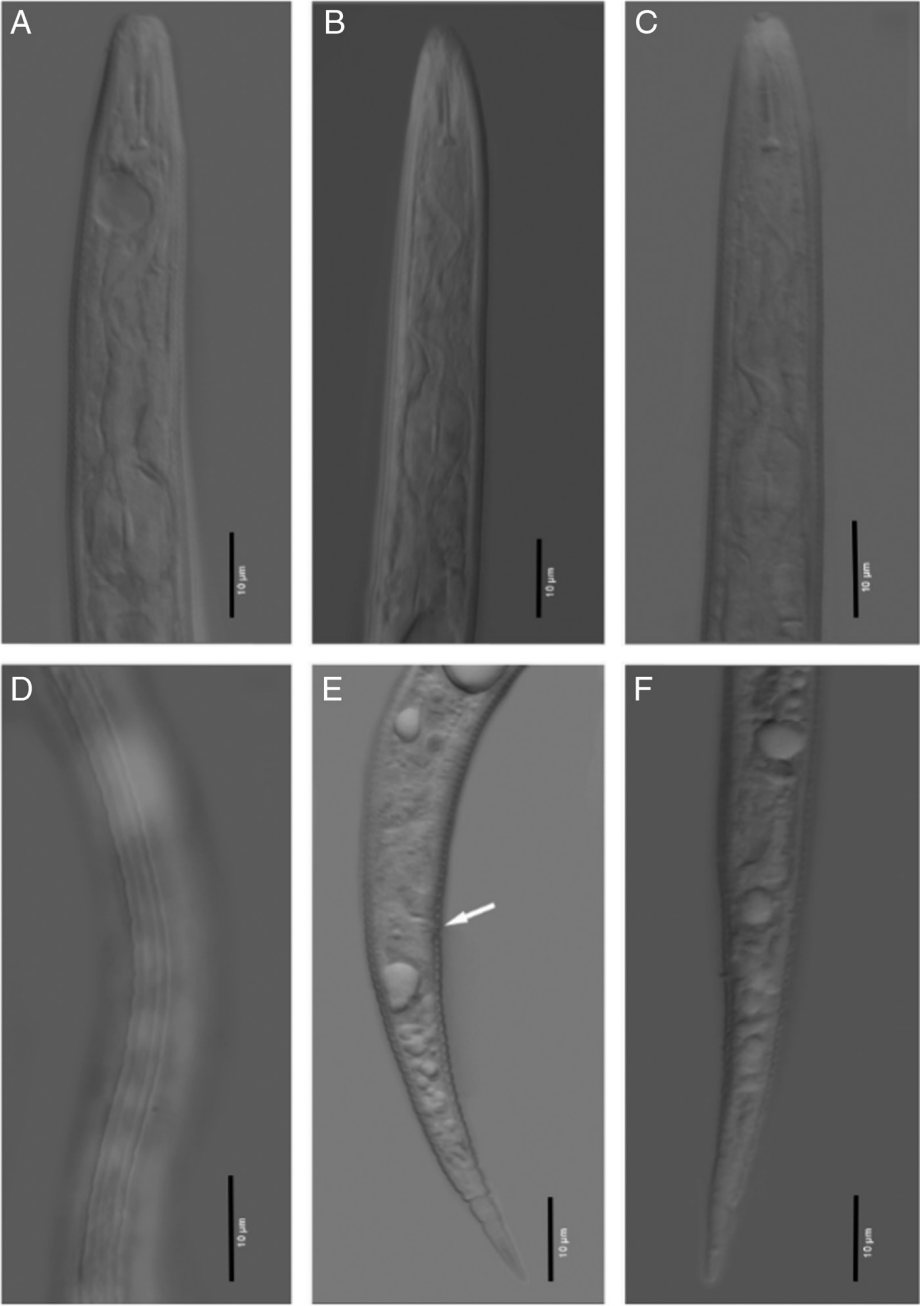
Photomicrographs of *Meloidogyne hipsanica* juveniles. (A–C) anterior end; (D) lateral field; (E, F) posterior ends with arrow pointing the anal area (E).

From *Meloidogyne luci* (Carneiro et al., 2014) juveniles are different by having a shorter stylet length (10.0–11.5 vs 12–13.5 μm) and by the shape of the tail and tail terminus which slender, with a bluntly rounded terminus vs conoid tail with finely rounded unstriated terminus.

### Molecular characterization

#### The partial *COI* gene

The partial *COI* gene sequence of *M. hispanica* was identical to those of *M. hispanica* (JX683712, JX683713) from China (Wang *et al.,* unpublished), *M. javanica, M. incognita, M. arenaria, M. floridensis* and several other species. It differed in one nucleotide from *COI* sequence of *M. luci.*

### The partial *nad5* gene

The partial *nad5* gene sequence was identical to that of *M. arenaria* (H3) *M. inornata* and *M. ethiopica* (Janssen et al., 2016).

### The D2-D3 of 28S rRNA gene

**Figure 2:**
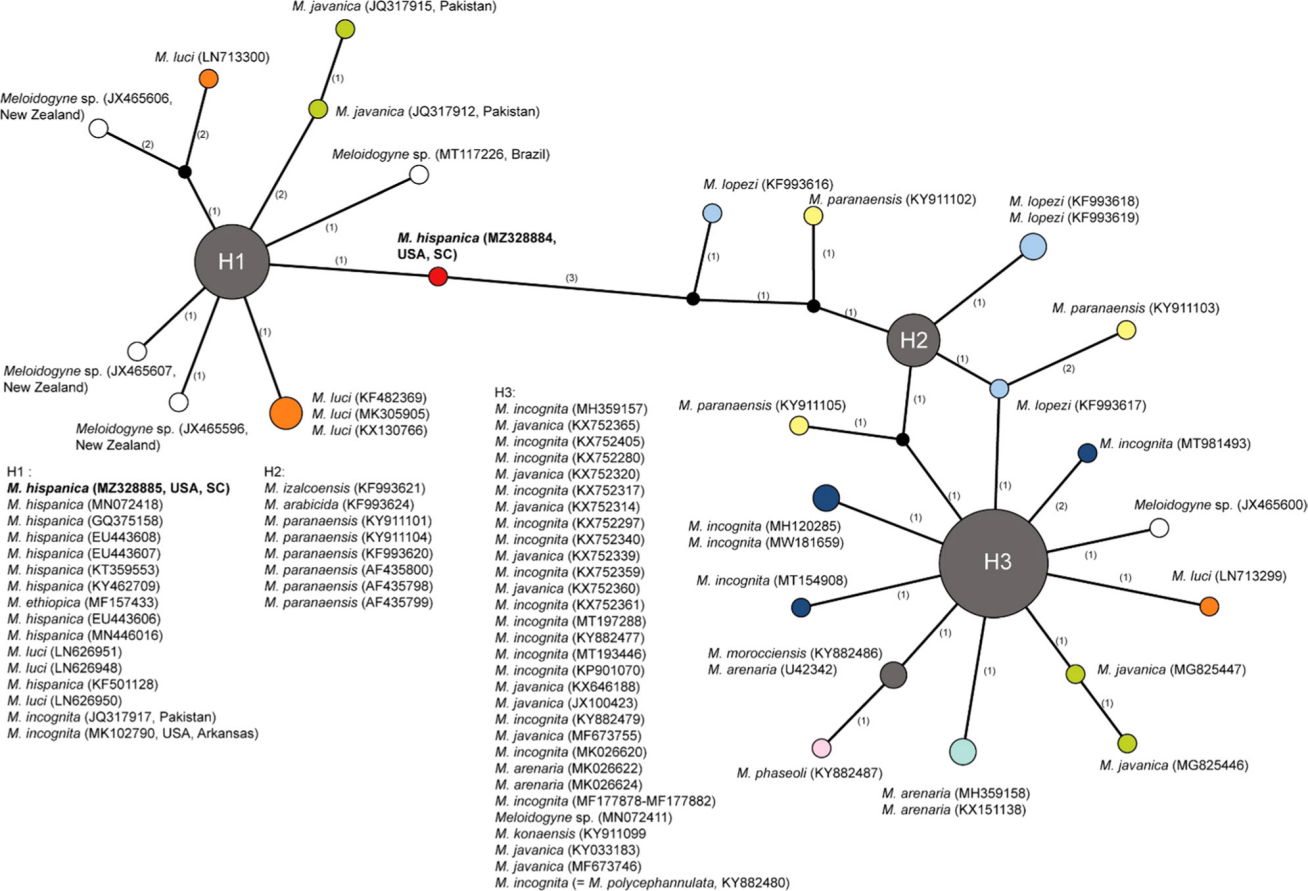
Statistical parsimony network showing the phylogenetic relationships between the D2–D3 of 28S rRNA gene sequences of *Meloidogyne* species from the tropical RKN complex. The sequences of each species are marked by different colors. Pies (circles) represent sequences of each species with the same haplotype and their size is proportional to the number of these sequences in the samples. Numbers of nucleotide differences between the sequences are indicated on lines connecting the pies. Small black dots represent missing haplotypes.

The D2–D3 of 28S rRNA gene alignment included 89 sequences and was 457 bp in length. The *M. hispanica* sequence from South Carolina (MZ328885) formed a separate group with other representatives of *M. hispanica, M. luci, M. ethiopica* and two sequences identified as *M. incognita* (Fig. 2).

### The ITS rRNA gene

**Figure 3:**
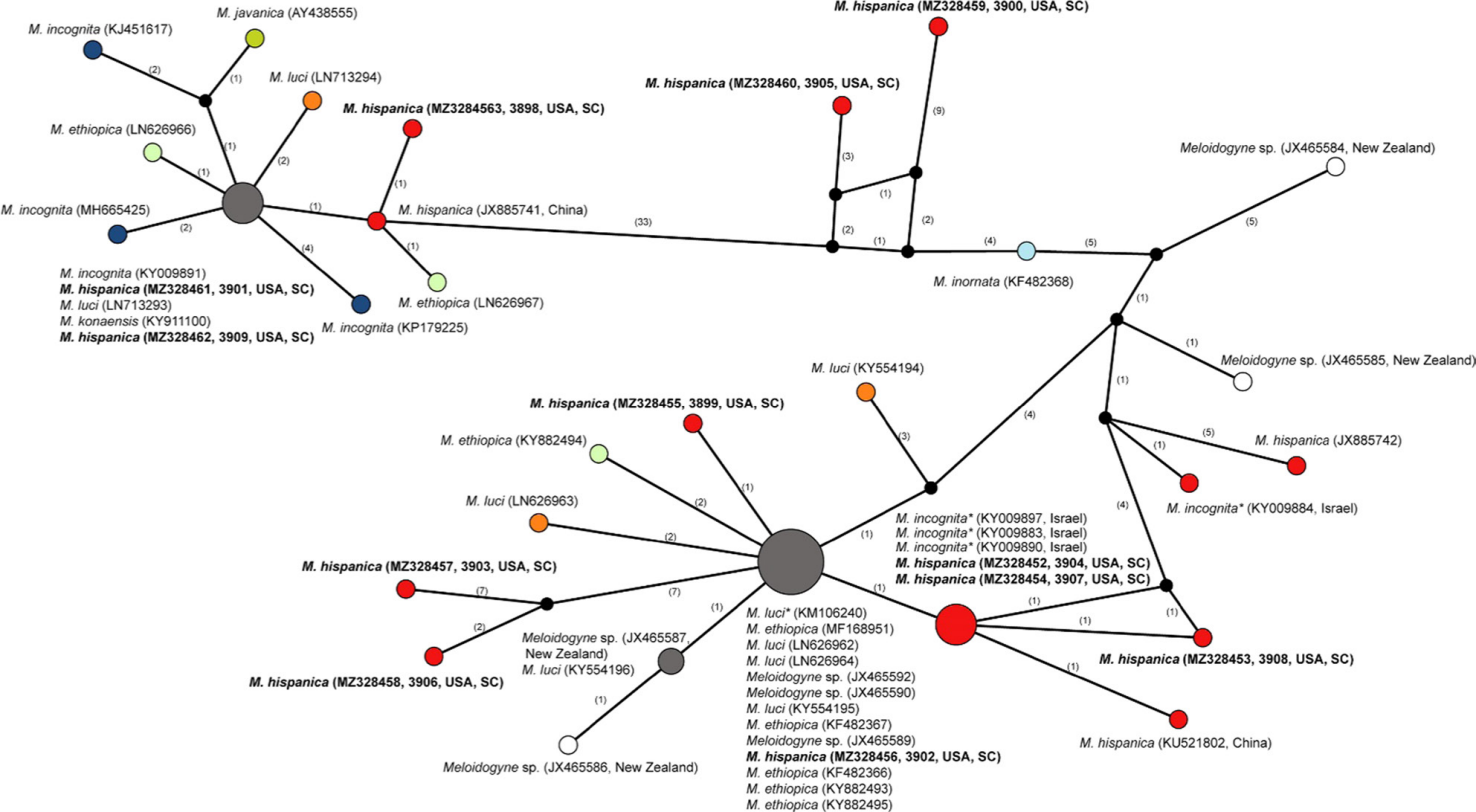
Statistical parsimony network showing the phylogenetic relationships between the ITS rRNA gene sequences of *Meloidogyne* species from the tropical RKN complex. The sequences of each species are marked by different colors. Pies (circles) represent sequences of each species with the same haplotype and their size is proportional to the number of these sequences in the samples. Numbers of nucleotide differences between the sequences are indicated on lines connecting the pies. Small black dots represent missing haplotypes. * - identified as *Meloidogyne incognita,* but maybe belong to *M. hispanica*.

The ITS rRNA gene alignment included 50 sequences and was 493 bp in a length. Twelve new sequences of the ITS rRNA gene clones from *M. hispanica* were distributed among the tropical RKN complex: (i) three sequences within the *Incognita* group; (ii) seven sequences within the *Ethiopica* group and two sequences formed a separate group (Fig. 3). Sequence variation between ITS rRNA gene clones from South Carolina *M. hispanica* reached 11.5%.

### The intergenic *COII*-16S gene region

**Figure 4:**
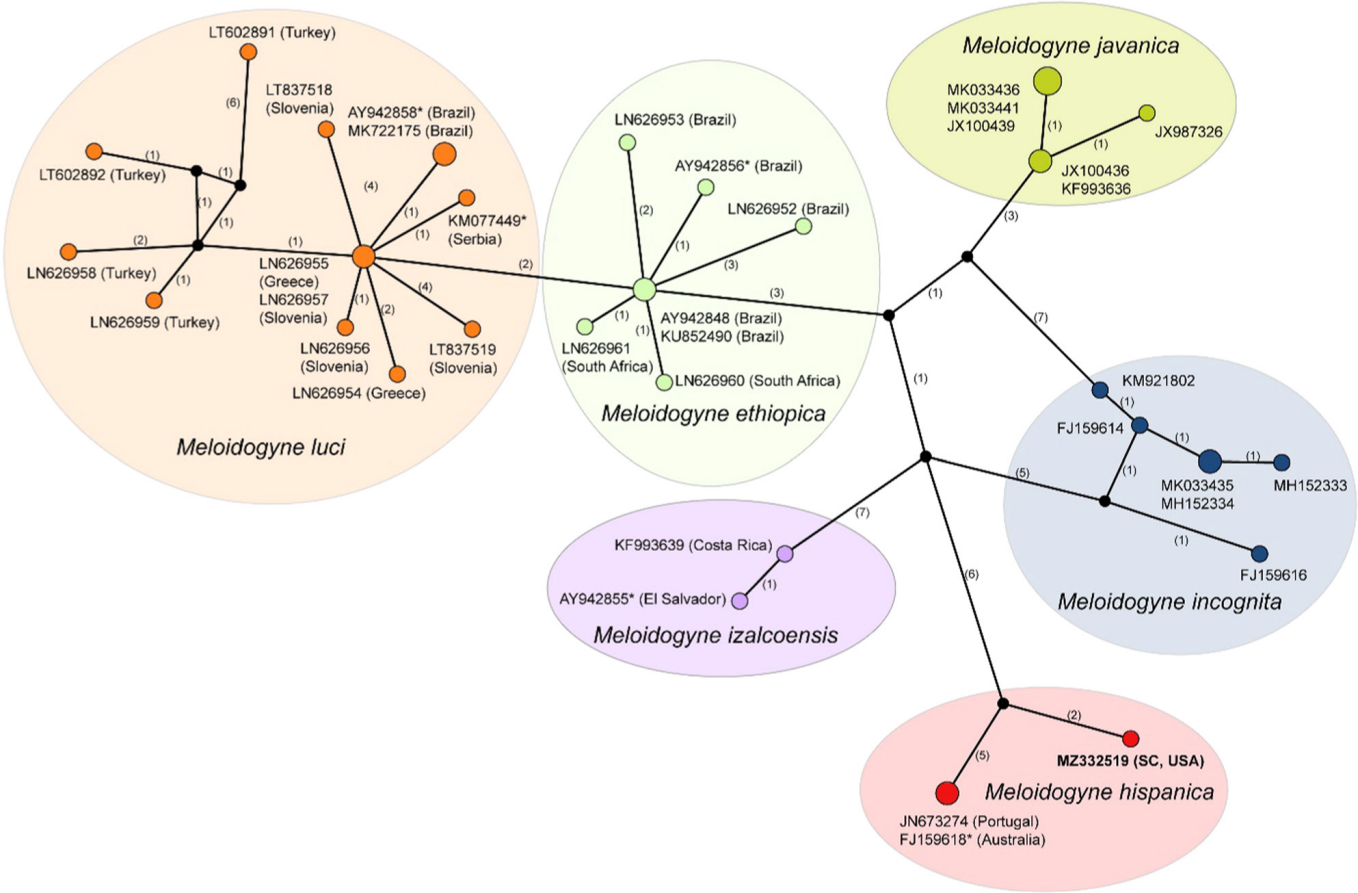
Statistical parsimony network showing the phylogenetic relationships between intergenic *COII*-16S gene sequences of *Meloidogyne* species having a long intergenic region. The sequences of each species are marked by different colors. Pies (circles) represent sequences of each species with the same haplotype and their size is proportional to the number of these sequences in the samples. Numbers of nucleotide differences between the sequences are indicated on lines connecting the pies. Small black dots represent missing haplotypes. * - identified in GenBank as *Meloidogyne* sp.

The *COII*-16S gene alignment included 37 sequences and was 1506 bp in length. Analysis of the intergenic region clearly differentiated *M. hispanica* from other related species (Fig. 4). The sequence of the South Carolina population clustered with sequence of *M. hispanica* from Portugal (Maleita et al., 2012a, 2012b) and sequence of *Meloidogyne* sp. from Australia (Fargette et al., 2010) identified here as a representative of this species.

Network analysis using statistical parsimony method was applied here for the study of relationships of root-knot nematodes. This method is especially useful for analysis of the datasets containing similar sequences. Moreover, the network can predict haplotypes and reveal sites where recombination and sequence errors are likely to have occurred. Since the network harbors all trees for the input data, it yields a more concise picture of relationships. Thus, our study showed that the ribosomal rRNA (ITS and D2–D3 of 28S) and mitochondrial *COI* and *nad5* gene sequences did not distinguish *M. hispanica* from other root-knot nematode species. Only the intergenic *COII*-16S gene region sequence allows clear separation of *M. hispanica* from closely related species.

To our knowledge the finding of *Meloidgyne hispanica* in South Carolina represents the first report of this species in the United States and North America. Reproduction of *M. hispanica* was previously evaluated on 63 cultivated host plants, revealing a broad host range (Maleita et al., 2012a, 2012b). *Meloidogyne hispanica* was most recently reported on corn in Greece in 2017 (Tzortzakakis et al., 2019) and on sunflower in Greece in 2013 (Tzortzakakis et al., 2014). The threat of damage caused by *M. hispanica* to corn or other susceptible crops in the U.S. remains to be determined but could become significant if present under drought conditions. Some degree of control may be achievable through rotation with pepper cultivars containing resistance genes (Maleita et al., 2012a, 2012b). Continued monitoring to limit further spread of *M. hispanica* is needed.

## References

[R1] Alvarez-Ortega, S. , Brito, J. A. and Subbotin, S. A. 2019. Multigene phylogeny of root-knot nematodes and molecular characterization of *Meloidogyne nataliei* Golden, Rose & Bird, 1981 (Nematoda: Tylenchida). Scientific Report 9:11788. 10.1038/s41598-019-48195-0 PMC6692364 31409860

[R2] Bandelt, H. , Forster, P. and Röhl, A. 1999. Median-joining networks for inferring intraspecific phylogenies. Molecular Biology and Evolution 16:37–48, doi: 10.1093/oxfordjournals.molbev.a026036. 10331250

[R4] Carneiro, R. M. D. G. , Correa, V. R. , Almeida, M. R. A. , Gomes, A. C. M. M. , Deimi, A. M. , Castagnone-Sereno, P. and Karssen, G. 2014. *Meloidogyne luci* n. sp. (Nematoda: Meloidogynidae), a root-knot nematode parasitising different crops in Brazil, Chile and Iran. Nematology 16:289–301.

[R5] Chenna, R. , Sugawara, H. , Koike, T. , Lopez, R. , Gibson, T. J. , Higgins, D. G. and Thompson, J. D. 2003. Multiple sequence alignment with the Clustal series of programs. Nucleic Acids Research 31:3497–3500. 12824352 10.1093/nar/gkg500 PMC168907

[R6] Dalmasso, A. and Bergé, J. B. 1978. Molecular polymorphism and phylogenetic relationship in some Meloidogyne spp.: application to the taxonomy of Meloidogyne Journal of Nematology 10:323–332. PMC2617902 19305862

[R8] De Ley, P. , Tandingan De Ley, I. , Morris, K. , Abebe, E. , Mundo-Ocampo, M. , Yoder, M. , Heras, J. , Waumann, D. , Rocha-Olivares, A. , Burr, A. H. J. , Baldwin, J. G. and Thomas, W. K. 2005. An integrated approach to fast and informative morphological vouchering of nematodes for applications in molecular barcoding. Philosophical Transactions of the Royal Society B 360:1945–1958. 10.1098/rstb.2005.1726 PMC1609217 16214752

[R9] Derycke, S. , Vanaverbeke, J. , Rigaux, A. , Backeljau, T. and Moens, T. 2010. Exploring the use of cytochrome oxidase c subunit 1 (COI) for DNA barcoding of free-living marine nematodes. PLoS ONE 5:1–9. 10.1371/journal.pone.0013716 PMC2965665 21060838

[R10] Fargette, M. , Berthier, K. , Richaud, M. , Lollier, V. , Franck, P. , Hernandez, A. and Frutos, R. 2010. Crosses prior to parthenogenesis explain the current genetic diversity of tropical plant-parasitic *Meloidogyne* species (Nematoda: Tylenchida). Infection, Genetics and Evolution 10:807–814. 10.1016/j.meegid.2009.04.013 19393769

[R11] Golden, A. M. 1990. “Preparation and mounting nematodes for microscopic observations”, Zuckerman, B. M. , Mai, W. F. and Krusberg, L. R. Plant Nematology Laboratory Manual. University of Massachusetts Agricultural Experiment Station, Amherst, MA, pp. 197–205.

[R12] Hirschmann, H. 1986. *Meloidogyne hispanica* n. sp. (Nematoda: Meloidogynidae), the’Seville root-knot nematode’. Journal of Nematology 18:520. 19294222 PMC2618593

[R14] Hooper, D. J. 1970. “Handling, fixing, staining, and mounting nematodes”. Southey, J. F. Laboratory Methods for Work with Plant and Soil Nematodes, 5th Edi., Her Majesty’s Stationery Office, London, pp. 39–54.

[R16] Janssen, T. , Karssen, G. , Verhaeven, M. , Coyne, D. and Bert, W. 2016. Mitochondrial coding genome analysis of tropical root-knot nematodes (*Meloidogyne*) supports haplotype- based diagnostics and reveals evidence of recent reticulate evolution. Scientific Reports 6:1–13. 26940543 10.1038/srep22591 PMC4778069

[R17] Jenkins, W. R. 1968. A rapid centrifugal flotation technique for separating nematodes from soil. Plant Disease Reporter 48:692.

[R18] Maleita, C. M. N. , Curtis, R. H. C. , Powers, S. J. and Abrantes, I. 2012a. Host status of cultivated plants to *Meloidogyne hispanica*. European Journal of Plant Pathology 133:449–460.

[R19] Maleita, C. M. , Simões, M. J. , Egas, C. , Curtis, R. H. and de O. Abrantes, I. M. 2012b. Biometrical, biochemical, and molecular diagnosis of Portuguese *Meloidogyne hispanica* isolates. Plant Disease 96:865–874. 30727353 10.1094/PDIS-09-11-0769-RE

[R21] Powers, T. O. and Harris, T. S. 1993. A polymerase chain reaction method for identification of five major *Meloidogyne* species. Journal of Nematology 25:1–6. 19279734 PMC2619349

[R23] Skantar, A. M. , Handoo, Z. A. , Zanakis, G. N. and Tzortzakakis, E. A. 2012. Molecular and morphological characterization of the corn cyst nematode, *Heterodera zeae*, from Greece. Journal of Nematology 44:58–66. 23482617 PMC3593258

[R25] Subbotin, S. A. , Palomares-Rius, J. E. , Castillo, P. 2021. Systematics of Root-Knot Nematodes (Nematoda: Meloidogynidae). Hunt, D. J. and Perry, R. N. Nematology Monograps and Perspectives Volume 14. Leiden, The Netherlands: Brill. (in press).

[R27] Thomas, W. K. , Vida, J. T. , Frisse, L. M. , Mundo, M. and Baldwin, J. G. 1997. DNA sequences from formalin-fixed nematodes: Integrating molecular and morphological approaches to taxonomy. Journal of Nematology 29:250–254. 19274156 PMC2619797

[R28] Tylka, G. L. , Sisson, A. J. , Jesse, L. C. , Kennicker, J. and Marett, C. C. 2011. Testing for plant-parasitic nematodes that feed on corn in Iowa 2000-2010. Plant health progress 12:2.

[R29] Tzortzakakis, E. A. , Anastasiadis, A. I. , Simoglou, K. B. , Cantalapiedra-Navarrete, C. , Palomares-Rius, J. E. and Castillo, P. 2014. First report of the root-knot nematode, *Meloidogyne hispanica*, infecting sunflower in Greece. Plant Disease 98:703. 30708522 10.1094/PDIS-08-13-0833-PDN

[R30] Tzortzakakis, E. A. , Cantalapiedra-Navarrete, C. , Archidona-Yuste, A. , Kormpi, M. , Palomares-Rius, J. E. and Castillo, P 2019. First report of cultivated Cretan mountain tea (*Sideritis syriaca*) as a host of *Meloidogyne hapla* and *M. javanica* in Crete, with some additional records on the occurrence of *Meloidogyne* species in Greece. Journal of Nematology 51e2019–10 https://dx.doi.org/10.21307%2Fjofnem-2019-010. 10.21307/jofnem-2019-010 PMC6929662 31088022

[R31] USDA. National Agricultural Statistics Service. Corn is America’s Largest Crop in 2019a. Available at: https://www.usda.gov/media/blog/2019/07/29/corn-americas-largest-crop-2019.

[R32] USDA. National Agricultural Statistics Service 2019b. State Agriculture Overview. Available at: https://www.nass.usda.gov/Quick_Stats/Ag_Overview/stateOverview.php?state=SOUTH%20CAROLINA.

[R33] Yan, G. , Friskop, A. and Ransom, J. 2016. Plant-Parasitic Nematodes on Corn and Evaluation of Corn Varieties for Resistance to Nematodes. Available at: https://www.ndcorn.org/plant-parasitic-nematodes-corn-evaluation-corn-varieties-resistance-nematodes/.

